# CpG island methylation is a common finding in colorectal cancer cell lines

**DOI:** 10.1038/sj.bjc.6600699

**Published:** 2003-02-10

**Authors:** C M Suter, M Norrie, S L Ku, K F Cheong, I Tomlinson, R L Ward

**Affiliations:** 1 Department of Medical Oncology, St Vincent's Hospital, Victoria St, Darlinghurst, NSW 2010, Australia; 2 School of Medicine, University of New South Wales, Sydney, NSW 2052, Australia; 3 Molecular and Population Genetics Laboratory, Cancer Research UK, London WC2A 3PX, UK

**Keywords:** colorectal carcinoma, methylation, microsatellite instability, CpG island

## Abstract

Tumour cell lines are commonly used in colorectal cancer (CRC) research, including studies designed to assess methylation defects. Although many of the known genetic aberrations in CRC cell lines have been comprehensively described, no studies have been performed on their methylation status. In this study, 30 commonly used CRC cell lines as well as seven primary tumours from individuals with hereditary nonpolyposis colorectal cancer (HNPCC) were assessed for methylation at six CpG islands known to be hypermethylated in colorectal cancer: *hMLH1*, *p16*, methylated in tumour (MINT-)-1, -2, -12 and -31. The cell lines were also assessed for microsatellite instability (MSI), ploidy status, hMLH1 expression, and mutations in APC and Ki*-ras*. Methylation was frequently observed at all examined loci in most cell lines, and no differences were observed between germline-derived and sporadic cell lines. Methylation was found at MINT 1 in 63%, MINT 2 in 57%, MINT 12 in 71%, MINT 31 in 53%, p16 in 71%, and *hMLH1* in 30% of cell lines. Overall only one cell line, SW1417, did not show methylation at any locus. Methylation was found with equal frequency in MSI and chromosomally unstable lines. MSI was over-represented in the cell lines relative to sporadic CRC, being detected in 47% of cell lines. The rate of codon 13 Ki-*ras* mutations was also over three times that expected from *in vivo* studies. We conclude that CpG island hypermethylation, whether acquired *in vivo* or in culture, is a ubiquitous phenomenon in CRC cell lines. We suggest that CRC cell lines may be only representative of a small subset of real tumours, and this should be taken into account in the use of CRC cell lines for epigenetic studies.

It is generally accepted that colorectal cancer (CRC) usually develops via one of two pathways–chromosomal instability (Kinzler and Vogelstein, 1996) or microsatellite instability (MSI) (Ionov *et al*, 1993). The molecular genetics of both these pathways are among the best understood of all human cancers. Recently, a third classification of CRC has emerged–the CpG island methylator phenotype (CIMP) (Toyota *et al*, 1999a). This classification is primarily based on the methylation status of certain sites in the genome, termed MINT loci, which are preferentially methylated in tumours. CIMP tumours are a subset of CRC with distinct clinicopathological characteristics and have been found to be associated with MSI (Ahuja *et al*, 1997; Hawkins *et al*, 2002).

Cell lines are widely used in CRC research, including studies on methylation, and are generally thought to be representative of the disease. Several groups have studied many commonly used cell lines at the genetic level, and generally the genetic changes in cell lines do reflect the changes seen *in vivo* (Ilyas *et al*, 1997; Rowan *et al*, 2000; Gayet *et al*, 2001; Woodford-Richens *et al*, 2001). However, despite the increased use of colorectal cell lines for epigenetic studies, there have been no comprehensive studies of the propensity for methylation in these cell lines.

In this study, we have examined the methylation status of four informative MINT loci, and the *p16* and *hMLH1* promoter, in 30 commonly used CRC cell lines. The aim of this study was to determine the frequency of CpG island hypermethylation in these cell lines, and determine if the epigenetic profile of these cell lines reflects that seen in primary colorectal tumours.

## MATERIALS AND METHODS

Methylation frequency in the set of sporadic tumours reported in the analysis was determined in a previous study of 417 prospectively collected tumours (Hawkins *et al*, 2002). Also included in the current analysis were seven primary tumours from seven individuals with a proven germline mutation in a mismatch repair gene (five *hMSH2* and two *hMLH1*). These primary tumours were collected as fresh representative tissue samples from consenting individuals undergoing surgical resection at the St Vincent's Campus. The 30 human colorectal cell lines examined in this study are listed in Table 1

**Table 1:** Genetic and epigenetic changes in 30 CRC cell lines

						**Methylation**						
**Cell line**	**MSI status**	**APC defect**	**K-ras mutation**	**Ploidy**	**MLH1 expression**	**MLH1-A**	**MLH1-C**	**p16**	**MINT-1**	**MINT-2**	**MINT-12**	**MINT-31**
SW620	MSS	Unknown	Codon 12	Aneuploid	Positive	U	U	M	U	P	U	U
C170	MSS	Unknown	None found	Diploid	Positive	U	U	NA	P	U	P	M
COLO741	MSS	None found	None found	Unknown	ND	NA	U	NA	U	M	U	U
COLO205	MSS	Biallelic	None found	Aneuploid	Positive	U	U	M	P	M	P	P
SW1116	MSS	Biallelic	Codon 12	Unknown	ND	U	NA	M	P	U	NA	U
SW1417	MSS	Biallelic	Codon 13	Aneuploid	ND	U	U	U	U	U	U	U
SW403	MSS	Biallelic	Codon 12	Aneuploid	Positive	M	U	U	U	U	U	U
SW480	MSS	Biallelic	Codon 12	Aneuploid	Positive	U	U	M	U	M	NA	U
SKCO1	MSS	Biallelic	Codon 12	Aneuploid	ND	M	U	U	P	U	U	P
SW1222	MSS	Biallelic	Codon 13	Aneuploid	ND	U	U	U	U	M	P	P
COLO320DM	MSS	Biallelic	None found	Diploid	Positive	U	U	M	U	U	P	U
SW948	MSS	Biallelic	None found	Aneuploid	ND	U	U	M	P	U	P	U
T84	MSS	Biallelic	Codon 13	Aneuploid	Positive	U	U	M	P	U	P	U
CACO2	MSS	Biallelic	None found	Aneuploid	Positive	U	U	M	P	P	P	P
HT29	MSS	Biallelic	None found	Aneuploid	Positive	U	U	M	P	P	P	P
SW837	MSS	Biallelic	Codon 12	Aneuploid	ND	U	NA	M	P	P	P	P
LIM1215^a^	MSI	Unknown	Codon 13	Diploid	Negative	U	U	U	U	U	U	P
LIM2412	MSI	Unknown	None found	Diploid	Negative	P	P	M	U	U	P	U
SNU-C2B	MSI	Unknown	Codon 12	Unknown	Positive	U	P	U	P	P	P	P
HCT8	MSI	Unknown	Codon 13	Diploid	Positive	U	U	M	P	P	P	U
RKO	MSI	Unknown	None found	Unknown	Negative	M	M	M	P	P	P	P
LS174T^a^	MSI	None found	Codon 12	Diploid	Negative	M	M	U	U	P	U	U
HCA7	MSI	None found	None found	Diploid	ND	M	M	M	U	U	U	U
SW48	MSI	None found	None found	Diploid	Negative	M	M	M	P	P	M	P
HCT116^a^	MSI	None found	Codon 13	Aneuploid	Negative	U	U	P	P	P	P	P
GP2D^a^	MSI	Biallelic	Codon 12	Diploid	ND	U	U	U	P	M	U	U
HCT15N^b^	MSI	Biallelic	Codon 13	Aneuploid	Positive	U	U	M	U	P	P	P
LOVO^c^	MSI	Biallelic	Codon 13	Diploid	Positive	U	U	M	P	U	P	P
LS411^a^	MSI	Biallelic	None found	Diploid	Negative	P	P	M	P	U	P	P
DLD-1^b^	MSI	Biallelic	Codon 13	Diploid	Positive	U	U	M	P	P	P	P

a MLH1 germline mutation.

b MSH6 germline mutation. MSS=microsatellite stable; MSI=microsatellite instability; M=methylated; U=unmethylated; P=partially methylated; NA=not amplifiable; ND=not done.

c MSH2 germline mutation.

. All cell lines were either originally derived from the ATCC, or received as kind gifts. Cells were cultured in DMEM, EMEM or RPMI, all supplemented with 10% foetal calf serum, and were harvested when growing exponentially. DNA was extracted from fresh tissue and cell lines using standard methods.

We have reported the APC status of each cell line previously (Rowan *et al*, 2000). Mutations in the first and second bases of codon 12 of Ki*-ras* were detected by REMS-PCR as described (Ward *et al*, 1998). An RFLP method was developed for the detection of Ki-*ras* codon 13 mutations. Genomic DNA (500 ng) was used in PCR with 0.5 *μ*M of each of the primers 5′-ATATAAACTTGTGGTAGTTCCAGCTGGT-3′ and 5′-ATCAAAGAATGGTCCTGCACC-3′, 2.5 mM MgCl_2_, 100 *μ*M dNTPs, 1.25 U FastStart TaqDNA polymerase (Roche Diagnostics GmBH, Mannheim, Germany) in the reaction buffer provided by the manufacturer. PCR cycling conditions were as follows: 95°C for 5 min; 10 cycles of 95°C for 20 s, 70°C for 20 s with a touchdown of 1°C per cycle; followed by a further 32 cycles of 95°C for 20 s, 60°C for 20 s, 72°C for 30 s prior to a final extension at 72°C for 4 min. Restriction fragment length polymorphism (RFLP) analysis was performed on amplicons at 55°C for 16 h with 10 U of *Bsl*I (NEB) according to the manufacturer's instructions. The resistance of PCR amplicons to *Bsl*I digestion indicated the presence of a mutation at the first or second base of Ki-*ras* codon 13.

The presence of MSI in each cell line was determined essentially as described (Hawkins and Ward, 2001) using primer sets for the pseudomonomorphic markers Bat25, Bat26 and Bat40 (Boland *et al*, 1998).

Assessment of ploidy was performed using the method of Taylor (1980). Briefly, washed tumour cells (2.5×10^6^) were incubated at 4°C for 10 min with a 1% Triton X-100 solution containing 100 *μ*g ml^−1^ of propidium iodide (Sigma, St. Louis, USA) in PBS. Cells were filtered through a nylon mesh to remove any clumps and incubated with DNAse-free RNAse for 30 min at 4°C before analysis on the flow cytometer.

For methylation analyses, DNA was treated with sodium bisulphite and methylation-specific PCR (MSP) was performed to detect methylation of the *p16* promoter region (Herman *et al*, 1998; Hawkins *et al*, 2002). The methylation status of MINT 1, 2, 12 and 31 was performed according to the method of Toyota *et al* (1999a), and modified as previously described (Hawkins *et al*, 2002).

Methylation of *hMLH1* was examined by bisulphite-RFLP at two separate regions of the *hMLH1* promoter (A and C) purported to be associated with *hMLH1* silencing (Herman *et al*, 1998; Deng *et al*, 2002). The A region, encompassing base pairs 801–1050 of the *hMLH1* promoter, was amplified with the primers MLH-AF, 5′-TTAYGGGTAAGTYGTTTTGAYGTAGA-3′ and MLH-AR, 5′-CCTATACCTAATCTATCRCCRCCTCA-3′. The C region, encompassing base pairs 1201–1450, was amplified with the primers MLH-CF, 5′-GGTTGGATATTTYGTATTTTTYGAG-3′ and MLH-CR, 5′-AATTACTAAATCTCTTCRTCCCTCC-3′. PCR was performed with 100 ng of bisulphite-modified template with 1 *μ*m of each primer, 1.5 mM MgCl_2_, 250 *μ*M dNTPs and 1.5 U FastStart TaqDNA polymerase (Roche Diagnostics GmBH, Mannheim, Germany) in the recommended buffer. PCR cycling conditions were as follows: 95°C for 5 min; 35 cycles of 95°C for 30 s, 55°C for 45 s, 72°C for 30 s before a final extension at 72°C for 4 min. The primers were designed to amplify both methylated and unmethylated template in the same reaction. Amplicons from methylated or unmethylated template were distinguished by restriction enzyme digestion with 10 U of *BstU*1. Amplicons from unmethylated template will not cut; amplicons from methylated template will be digested. MLH1 expression was determined in parallel, in a subset of 20 cell lines, by Western blot, or immunostaining, as previously described (Ward *et al*, 2001; Li *et al*, 2002).

Methylation was reported for each locus as unmethylated, partially methylated or methylated. For *p16*, partial methylation was indicated by amplification in both the unmethylated and methylated MS-PCR reactions. For the MINT loci and *hMLH1*, partial methylation was recorded when the digestion of PCR products was incomplete. For *hMLH1*, the A and C regions were considered as one locus for analysis. Positive and negative controls were included in each procedure, and neither control revealed partial methylation in any assay. PCR reactions for all loci were performed from the same bisulphite-treated reaction in the majority of cases.

Categorical variables were compared using the *χ*^2^ test or the Fisher exact test as appropriate. A probability value of <0.05 was considered statistically significant. Statistical analysis was performed with SPSS statistical software V9.0 (SPSS Inc., Chicago, IL, USA).

## RESULTS

The genetic and epigenetic changes in each cell line are detailed in Table 1. Overall, 87% (24 out of 30) of cell lines demonstrated methylation at two or more than six loci. The median number of methylated sites was 3, and this was observed in 44% of cell lines. Methylation at all six loci was seen in 7%. The majority of primary tumours from HNPCC individuals displayed virtually no CpG island hypermethylation; 6 out of 7 had less than two of the loci methylated (Table 2

**Table 2:** Methylation profile of seven HNPCC patients

			**Methylation**			
**HNPCC patient**	**MLH1-A**	**p16**	**MINT1**	**MINT2**	**MINT12**	**MINT31**
1^a^	U	U	M	U	U	U
2^a^	U	U	U	U	U	U
3^b^	U	U	U	U	U	U
4^b^	U	U	U	U	U	U
5^b^	U	U	U	U	U	U
6^b^	M	M	M	M	U	U
7^b^	M	U	U	U	U	U

a hMLH1 germline.

b hMSH2 germline.

). There were no significant differences, however, in the methylation propensity of sporadic cell lines *vs* germline-derived cell lines. The frequency of hypermethylation at individual loci in both types, compared with primary tumours, is shown in Table 3

**Table 3:** Frequency of methylation at individual loci

**Loci**	**Sporadic CRC^a^ *n*=417 (%)**	**Cell line total *n*=30**	**HNPCC *n*=7**	**Cell line sporadic *n*=22**	**Cell line HNPCC *n*=8**
Mint-1	25	63% (18)	29% (2)	59% (13)	63% (5)
Mint-2	29	57% (17)	14% (1)	55% (12)	63% (5)
Mint-12	29	68% (19)	0% (0)	70% (14)	63% (5)
Mint-31	14	53% (16)	0% (0)	46% (10)	75% (6)
P16	37	71% (20)	14% (1)	75% (15)	63% (5)
MLH1	33	30% (9)	29% (2)	32% (7)	25% (2)

a Hawkins *et al* (2002).

. A significant increase in the frequency of methylation in cell lines compared to primary tumours is apparent (*P*<0.01), regardless of germline status.

**Figure 1:**
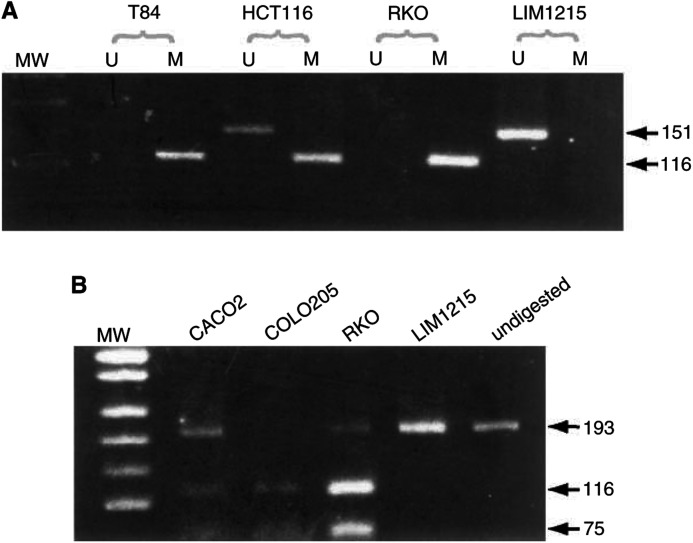
Analysis of methylation of *p16* and MINT loci in colorectal cell lines. (**A**) For *p16* promoter analysis, bisulphite-modified cell line DNA was amplified in separate reactions using primers specific for unmethylated (U) or methylated (M) template (methylation-specific PCR - MSP). Examples of methylated cell lines are T84 and RKO; these show amplification in the methylated reaction only. Presence of PCR product in both unmethylated and methylated reactions of HCT116 is indicative of partial methylation at *p16*. LIM1215 is shown as an example of a cell line unmethylated at *p16*. (**B**) For analysis of methylation at MINT loci, bisulphite-modified DNA was first PCR amplified using appropriate loci-specific primers, and then the PCR products were digested with restriction enzymes that only cut amplicons generated from the methylated template. MINT2 is shown as a representative MINT locus. Resistance to digestion indicates an unmethylated template. Shown are cell lines with partial methylation at MINT 2, CACO2 and RKO, a fully methylated cell line, COLO205, and an unmethylated cell line LIM1215. Undigested amplicons are shown in the last lane. Molecular weight (MW) marker in A and B is pUC19/*Msp*I.

Methylation of *p16* (Figure 1A

) was present in 71% (20/28) of cell lines assessable at this locus. One cell line, HCT116, showed partial methylation, and this has been shown previously to be because of the presence of a somatically mutated, but nonmethylated allele (Myohanen *et al*, 1998). Methylation at *p16* could not be reported in two cell lines, COLO741 and C170, owing to the lack of amplification of *p16*, and this is likely because of biallelic loss of *p16*.

Methylation at the MINT loci (Figure 1B) was also a frequent finding in most of the cell lines, and this was usually observed as partial methylation. Partial methylation was considered real, and not an artefact of the bisulphite reaction, as PCR reactions for all the examined loci were usually performed from the same bisulphite reaction. In addition, peripheral blood control DNA was included at each step, along with all samples. Furthermore, overdigestion with excess enzyme performed on a subset of these partially methylated samples showed the same result. The frequencies of MINT methylation ranged from 53 to 68%, and at all loci the frequency was significantly higher than that observed in sporadic CRC (Table 3). Like *p16*, the frequency of methylation of MINT loci was increased in the cell lines irrespective of germline status; only one HNPCC primary tumour displayed some significant methylation.

**Figure 2:**
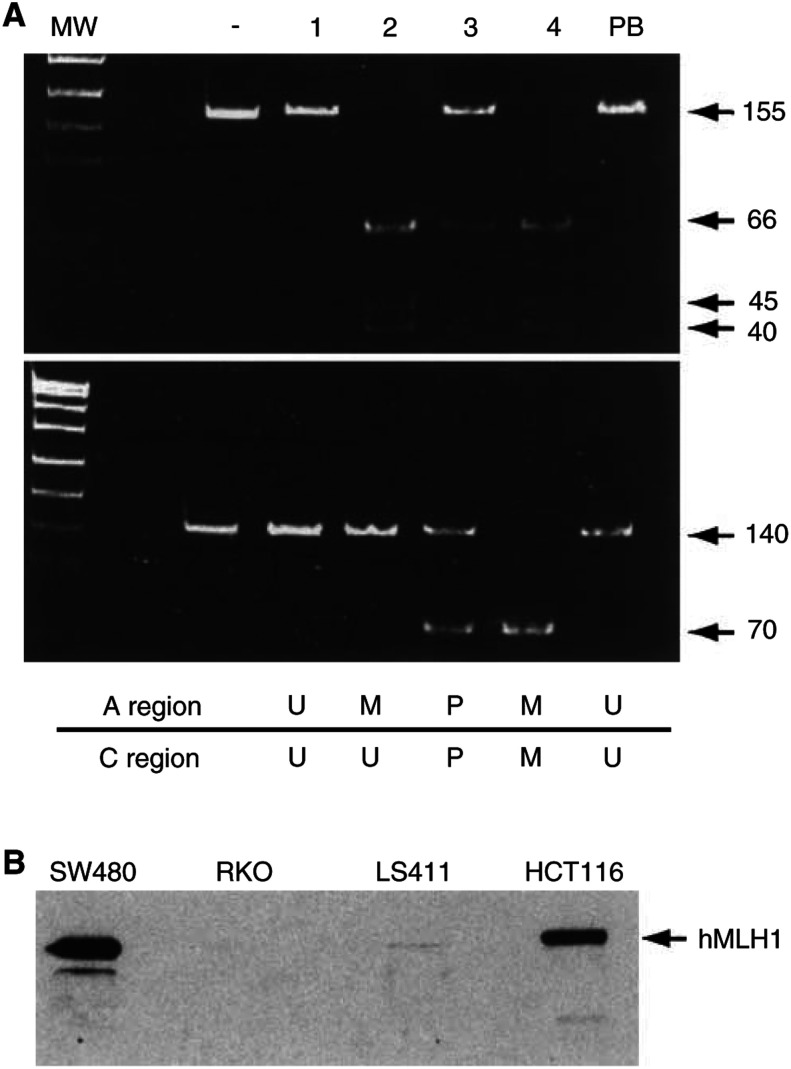
Analysis of *hMLH1* methylation and expression in colorectal cell lines. (**A**) Both the A region (top panel) and C region (bottom panel) of the *hMLH1* promoter were amplified from bisulphite-treated DNA. *Bst*U1 restriction enzyme digest was performed to distinguish amplicons from methylated or unmethylated template; BstU1 will cut only amplicons generated from methylated template. Undigested PCR product is shown in the control lane (-). Lane 1 shows an example of a cell line unmethylated at both the A and C regions, DLD1. Lane 2 shows SW403 that is methylated at the A region, but not C. Lane 3 shows LS411 that is partially methylated at both regions and Lane 4 shows a fully methylated cell line, RKO. Peripheral blood (PB) DNA used as a control is always unmethylated at both the A and C regions. MW market is pUC19/*Msp*I. (**B**) Representative Western blot analysis of hMLH1 expression in colorectal cell lines with various combinations of A and C region methylations. SW480 has no methylation at either region and has wild-type *hMLH1*. HCT116 also is unmethylated at both regions; however, this cell line has a frameshift mutation in one allele of *hMLH1*, leading to a reduction in expression levels. Cell lines exhibiting either full (RKO) or partial (LS411) methylation at both the A and C regions show a loss of hMLH1 protein.

Methylation of *hMLH1* at either the A or C region occurred in 30% of assessable cell lines (A, 28%; C, 25%), Figure 2A

. Either full or partial methylation of both regions was strongly associated with loss of hMLH1 expression (*P*<0.001), Figure 2B. Methylation of the A or C region alone did not affect the expression of MLH1. Only one cell line, LIM1215, showed a loss of hMLH1 expression in the absence of promoter methylation; however, this cell line has a germline *hMLH1* mutation, and probably LOH as the second hit (Whitehead *et al*, 1985).

There was only one cell line that showed no methylation at any of the six loci–SW1417. Although this unmethylated cell line was microsatellite stable (MSS), there was no significant association overall of MSI and the degree of methylation. There was no association between the methylation of any individual marker and the presence of MSI, with the exception of *hMLH1* (A or C region methylation, *P*=0.03). Apart from this expected association, methylation overall was found to occur with approximately equal frequency in MSS and MSI cell lines.

Microsatellite instability itself was detected in 14 cell lines (47%), and a biallelic inactivation of APC in 18 (60%). The two were not always mutually exclusive, with five cell lines (GP2D, HCT15N, LOVO, LS411 and DLD-1) showing both MSI and APC inactivation. As expected, the presence of MSI was highly associated with diploid cell lines (*P*=0.001) and APC mutants were more likely to be aneuploid (*P*=0.03). There was also a significant association between the presence of MSI and the loss of hMLH1 expression (*P*=0.03).

**Figure 3:**
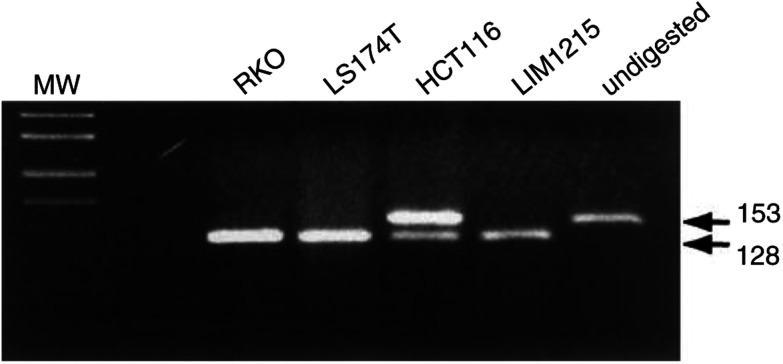
Detection of mutations in codon 13 of Ki-*ras* in colorectal cell lines. RFLP analysis was performed on Ki-*ras* PCR amplicons with the restriction enzyme *Bsl*I. Mutations in the first or second base of codon 13 will abolish the *Bsl*I site; therefore Ki-*ras* codon 13 mutants are resistant to digestion. Wild-type (WT) amplicons, and mutants other than codon 13, are fully digested by *Bsl*I. Undigested amplicons are shown in the last lane. Both LIM1215 and RKO have WT Ki-*ras*; LS174T is a codon 12 mutant; HCT116, displaying undigested product, is a codon 13 mutant. MW is pUC19/*Msp*I.

A remarkably high rate of Ki-*ras* mutations was found in this data set. Of the 30 cell lines, 19 (63%) were found to have a point mutation in either codon 12 or codon 13 of Ki*-ras* (Figure 3

). Codon 12 mutations occurred in 33%, and this is comparable to the expected frequency for CRC (Andreyev *et al*, 2001). The proportion of cell lines harbouring mutations in codon 13 of Ki-*ras* in these cell lines was 30%. This is considerably higher than the reported frequencies of around 8.6% (Andreyev *et al*, 2001).

## DISCUSSION

This study has revealed that methylation is very common in CRC cell lines. Methylation at most loci was observed at a significantly greater frequency than that expected from *in vivo* studies (Hawkins *et al*, 2002). The reason for the excessive levels of methylation in CRC cell lines as opposed to their primary counterparts is unclear.

Significantly lower levels of CpG island hypermethylation were observed in HNPCC primary tumours as compared to the sporadic tumours. This agrees with other reports on methylation in primary HNPCC tumours (Yamamoto *et al*, 2002); however, the cell lines do not parallel this difference. The propensity for hypermethylation in the cell lines did not differ between those derived from sporadic tumours, or HNPCC-derived tumours. The colorectal cell lines displayed significantly higher rates of methylation than that observed in primary CRC, whether it is sporadic, or associated with a germline mutation.

There may be several reasons for the high levels of methylation reported here. It is possible that it is acquired *in vitro* in a culture environment, which is in some way conducive to methylation. In support for this, one other study of methylation in 24 cell lines of various origins found that all of the cultured cell lines exhibited significantly more methylation at anonymous loci than the primary malignancies that they were derived from (Smiraglia *et al*, 2001). Using restriction genome landmark scanning (RLGS), Smiraglia and colleagues concluded, however, that the three colon cancer cell lines in their study were in fact the most similar to their primary counterparts. Other cell lines in this study, such as head and neck squamous cell carcinoma, had up to a 93-fold increase in hypermethylation of RLGS fragments. The authors conclude that most of the hypermethylation seen in cancer cell lines is because of an intrinsic property of cell lines, as opposed to the primary tumour from which they were derived. Tracking the epigenetic changes from individual tumours to their establishment as cell lines may distinguish between what, if any, is culture-induced methylation and what existed *in vivo*. Unfortunately, the most commonly used cell lines in CRC research have been established for over 20 years, and such epigenetic profiling is likely to be impossible now.

If methylation is occurring as a direct result of *in vitro* culture conditions, then it is likely to induce changes in the cells that render them nonrepresentative of the tumours from which they were derived. The tumour suppressor gene *p16* is commonly methylated in a wide variety of human primary tumours and cell lines (Herman *et al*, 1995; Merlo *et al*, 1995). Inactivation of *p16* allows the cancer cell to escape senescence, and the normal cell cycle controls, to rapidly proliferate. If methylation and inactivation were to occur *in vitro*, the result would be altered growth characteristics, as compared to the parent tumour. Likewise, while the majority of the MINT sequences have not been described as genes, they may have important, albeit unknown, functions. MINT31 (also known as CACNA1G) has been mapped to a location of frequent LOH in cancer, and was found to be part of a gene encoding a T-type calcium channel (Toyota *et al*, 1999b). Such genes involved in modulating calcium signalling are likely to be important in cancer cell proliferation and apoptosis. Genes with important functions are often the targets of hypermethylation in cancer, with consequences affecting many cellular pathways. Besides methylation of *p16* and CACNA1G, a high rate of methylation of other important genes is observed in CRC. These include *hMLH1* (Kane *et al*, 1997; Herman *et al*, 1998) (mismatch repair), *O*^*6*^*MGMT* (Esteller *et al*, 2000b) (DNA repair) and *p14/ARF* (Esteller *et al*, 2000a; Zheng *et al*, 2000) (p53 pathway). We have also found a high rate of methylation of *hMLH1* in the colon cancer cell lines although this was one marker that was not methylated at a higher rate in cell lines as opposed to primary tumours. It is possible that owing to the high rate of MSI in the cell lines, there is no selection pressure to methylate *hMLH1*. Methylation at both the A and C regions of the promoter was strongly related to loss of hMLH1 expression. This is consistent with recent reports that only dense methylation of the *hMLH1* promoter is associated with gene silencing and loss of protein expression (Furukawa *et al*, 2002). The presence of partial methylation at both the A and C regions was also seen in cell lines lacking hMLH1 expression (LS411). This suggests that the high rate of partial methylation seen at the MINT loci is also functionally important. The study of *p14* methylation by Zheng and colleagues also identified partial methylation of *p14* associated with reduced gene expression. Coincidentally, this study also revealed a greater percentage of methylated *p14* in cell lines (40%) as opposed to primary colon cancers (18%).

An alternative explanation for the high frequency of methylation observed in this study is that cell lines are more likely to be established from those tumours that are methylated *in vivo*. Tumour cell lines on the whole are difficult to establish, and perhaps the methylator type of tumour is more amenable or adaptable to culture conditions. Irrespective of the cause or timing of this aberrant methylation, there is evidence to suggest that it is important for CRC cell lines, and there is a strong selection pressure to maintain it. For example, many *in vitro* studies have shown that the methylation of various promoters is never 100% reversible. When cultured cells with CpG island methylation are exposed to demethylating 5-azacytidine treatments, methylation always returns upon withdrawal of the drug in a gene-specific manner, and has recently been shown to return, and be hereditable, in a parental allele-specific fashion (Li *et al*, 2002). This could argue that the hypermethylation present in these cell lines is not a phenomenon of culture, but rather a necessary and characteristic component of the cell line and the tumour from which it was derived.

Regardless of the cause of the excessive methylation in CRC cell lines, the end result is the same and CRC cell lines may only serve as a model for a subset of tumours. Considering that genes commonly methylated in CRC are often involved in cell proliferation, apoptosis and DNA repair, there is likely to be a very skewed bias of cell lines in many areas of research, not only methylation studies. A bias within these cell lines is further supported by the increased frequency of Ki-*ras* mutations and the over-representation of MSI.

MSI occurs at a frequency of 10–15% in sporadic CRC, but was observed at double the frequency in the sporadic cell lines. It was not surprising to find that the MSI cell lines in this study had a high frequency of methylation, as the association between the two phenomena has been well reported (Toyota *et al*, 1999a,^2000^; Hawkins *et al*, 2002). What was more surprising was the high frequency of methylation in MSS cell lines. The fact that methylation can coexist with APC mutations in sporadic CRC has been reported by others, albeit with a lesser frequency (Gayet *et al*, 2001; Hawkins *et al*, 2002). These findings argue against the hypothesis that methylation is a third distinct pathway of colorectal tumourigenesis, and in favour of it being a feature of both standard pathways. Whether promoter methylation is a cause or a consequence of either pathway cannot be determined by this study.

The high rate of Ki-*ras* codon 13 mutations in this study is also worthy of mention in terms of methylation. There is a reported association between Ki-*ras* mutations and highly methylated tumours (Toyota *et al*, 2000; Hawkins *et al*, 2002), however, codon 12 mutations are the predominating type in these studies of sporadic colorectal tumours. A high frequency of codon 13 mutations has only been reported once before, in a set of HNPCC tumours with MSI (Fujiwara *et al*, 1998). This is reflected in the germline-derived cell lines in this study; however, a significant proportion of codon 13 mutations also occurred in Ki-*ras* of nongermline cell lines. The increased frequency of codon 13 mutations in the cell lines, regardless of germline status, may in part be explained by a methylator phenotype. It has recently been shown that methylation of *O*^6^-MGMT associates with Ki-*ras* mutations in CRC (Esteller *et al*, 2000b; Whitehall *et al*, 2001). This is likely because of the increased rate of G to A transition mutations caused by the silencing, and subsequent deficiency, of *O*^6^-MGMT. It is also worthy of note that Ki-*ras* mutations have been found to associate with *p16* methylation in colorectal tumours and adenomas (Guan *et al*, 1999). These results together provide further evidence to support the notion that CRC cell lines are predominantly representative only of the methylator type of CRC.

Whether cause or consequence, epiphenomenon or true epigenetic change, the high frequency of methylation in these cell lines exists, and should be taken into account in studies on the biology of CRC. We would suggest that cell lines may be good models for the methylator type of CRC, and are not generally representative of sporadic CRC.
